# Cdh2 stabilizes FGFR1 and contributes to primed-state pluripotency in mouse epiblast stem cells

**DOI:** 10.1038/srep14722

**Published:** 2015-09-30

**Authors:** Toshiyuki Takehara, Takeshi Teramura, Yuta Onodera, John Frampton, Kanji Fukuda

**Affiliations:** 1 Division of Cell Biology for Regenerative Medicine, Institute of Advanced Clinical Medicine, Kindai University Faculty of Medicine, 377-2 Ohnohigashi, Osaka-sayama, Osaka, Japan 5898511; 2 School of Biomedical Engineering, Dalhousie University, Halifax, Nova Scotia, Canada B3H 4R2 1-902-494-4175

## Abstract

The cell adhesion molecule Cadherin 2 (Cdh2) plays important roles in somatic cell adhesion, proliferation and migration. Cdh2 is also highly expressed in mouse epiblast stem cells (mEpiSCs), but its function in these cells is unknown. To understand the function of Cdh2 in mEpiSCs, we compared the expression of pluripotency-related genes in mEpiSCs and mouse embryonic stem cells (mESCs) after either Cdh2 knockdown or Cdh2 over-expression. Introduction of specific siRNA against Cdh2 led to attenuation of pluripotency-related genes. Pluripotent gene expression was not recovered by over-expression of Cdh1 following Cdh2 knockdown. Western blot analysis and co-immunoprecipitation assays revealed that Cdh2 stabilizes FGFR1 in mEpiSCs. Furthermore, stable transfection of mESCs with Cdh2 cDNA followed by FGF2 supplementation accelerated cell differentiation. Thus, Cdh2 contributes to the establishment and maintenance of FGF signaling-dependent self-renewal in mEpiSCs through stabilization of FGFR1.

A complete understanding of the molecular network that regulates pluripotency will provide new insights on mammalian early development and accelerate the discovery of novel technologies for producing stem cells. To date, pluripotent stem cells have been established from mouse^1^,^2^, rat^3^, primate^4^ and human^5^ cell sources. There is significant phenotypic variability among these cell sources, as highlighted by the fact that mouse and human stem cells can exist in two different pluripotent states: naïve and primed^6^.

Mouse embryonic stem cells (mESCs) derived from the inner cell mass (ICM) of a blastocyst at 3.5 days post coitum (dpc) are typical naïve-state pluripotent stem cells. These mESCs form round dome-shaped colonies on mouse embryonic fibroblast (MEF) feeder layers and require LIF/STAT3 signaling to maintain pluripotency^7^. These naïve mESC can differentiate into many different fetal cell types including germ cells upon injection into mouse pre-implantation embryos. Naïve human pluripotent stem cells with similar properties were recently produced using specially modified culture conditions^8^,^9^,^10^,^11^,^12^,^13^. On the other hand, mouse epiblast stem cell (mEpiSC) derived from the epiblast of an embryo at 5.75 to 6.5 dpc are typical primed-state pluripotent stem cells^14^,^15^. Primed pluripotent stem cells, such as mEpiSCs and human ESCs, form flat colonies and undergo self-renewal by way of Activin/Nodal and basic FGF/Mek/Erk signaling. Naïve and primed pluripotent stem cells also express different cell surface glycoproteins, integrins and cadherins.

Analyzing cadherin expression in naïve mESCs and primed mEpiSCs represents an attractive starting point for unravelling key differences between naïve and primed pluripotent stem cells, because cadherins not only regulate stem cell colony morphology, but also contribute to important cellular events such as proliferation, migration and differentiation^16^. Cadherin1 (E-cadherin, epithelial-cadherin; Cdh1), which is the predominant cadherin expressed by mESCs, is thought to contribute to the compact cell morphology of mESCs^17^. Cdh1 is a single transmembrane glycoprotein with five extracellular domains that participate in calcium-dependent homophilic cell-cell adhesion^18^. The intracellular domain of Cdh1 interacts with the actin cytoskeletal through catenin proteins^19^. While Cdh1 expression is robust in mature epithelial cells, it is also appears during the compaction phase of mouse early embryonic development in morula stage embryos^20^. Interestingly, recent studies have shown that Cdh1 stabilizes STAT3-mediated signaling by binding to LIF/GP130 and subsequently activating pluripotency-related genes such as Nanog in mESCs^21^. These facts suggest that cadherins are involved in stem cell development.

We previously reported that Cadherin2 (N-cadherin, neuronal-cadherin; Cdh2) is the predominant cadherin expressed by mEpiSCs^22^. We also observed that the conversion from mESCs to mEpiSCs coincides with cadherin-switching from Cdh1 to Cdh2. However, the function of Cdh2 in mEpiSCs and the significance of cadherin-switching are still unknown. In this study, we investigate the expression status, function and significance of Cdh2 expression in mEpiSCs.

## Results

### Cdh2 is the predominant cadherin expressed by mEpiSCs

**Figure 1:**
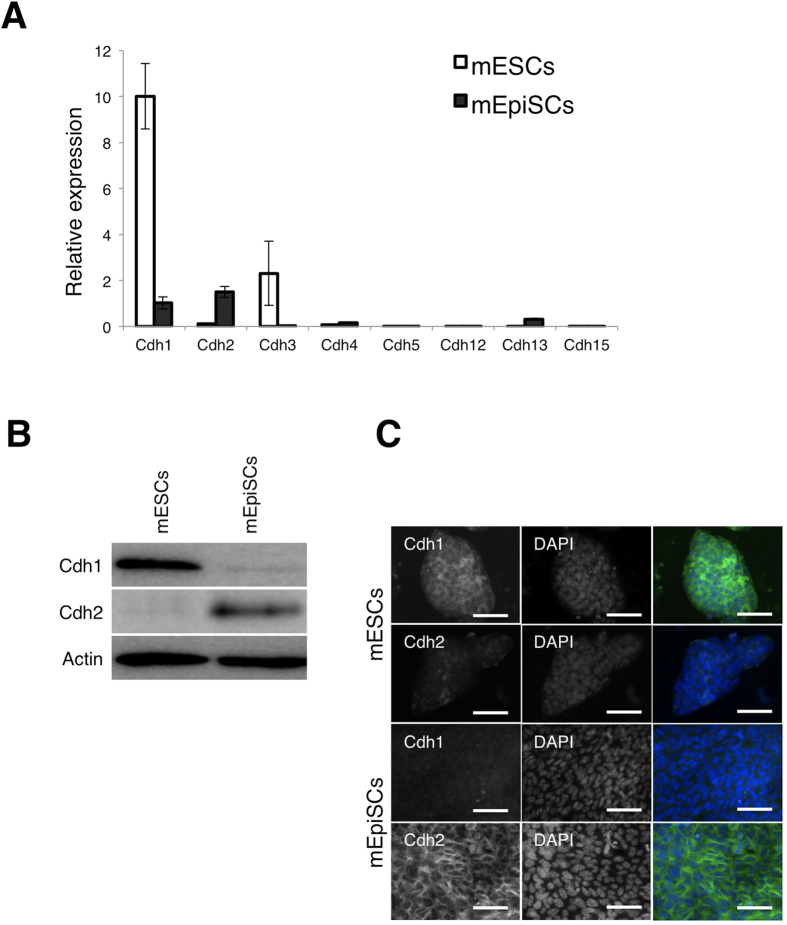
Cdh2 is the major cadherin-type expressed by mEpiSCs. (**A**) qRT-PCR analysis for classical and atypical cadherins in mESCs and mEpiSCs. Bars represent the mean values of triplicates. White bars indicate gene expression in the mESCs and gray bars indicate gene expression in the mEpiSCs. (**B**) WB analysis for Cdh1 and Cdh2 in mESCs and mEpiSCs. (**C**) Immunofluorescent staining for Cdh1 and Cdh2 in mESCs and mEpiSCs. The scale bars are 100 μm.

We first analyzed the expression of a variety of classical and atypical cadherin genes: *Cdh1* (Epithelial-cadherin), *Cdh2* (Neuronal-cadherin), *Cdh3* (Placental-cadherin), *Cdh4* (Retinal-cadherin), *Cdh5* (Vascular Endothelial-cadherin), *Cdh12* (Neuronal-cadherin II), *Cdh13* (T-cadherin, heart-cadherin) and *Cdh15* (Myotubular-cadherin) by quantitative RT-PCR (qRT-PCR). In mESCs, *Cdh1* was the most highly expressed of the cadherin genes, although *Cdh3* was also expressed. In contrast, mEpiSCs predominantly expressed *Cdh1* and *Cdh2* (Fig. 1A). We next determined Cdh1 and Cdh2 protein expression in mESCs and mEpiSCs by Western blot (WB) analysis (Fig. 1B). Cdh1 was abundant in mESCs, with only low levels of Cdh2. In contrast, Cdh2 was abundant in mEpiSCs, with almost no Cdh1. Immunofluorescence confirmed the expression of Cdh1 and Cdh2 in the mESCs and mEpiSCs, respectively (Fig. 1C). From these results, we conclude that Cdh1 is a mESC status-specific cadherin and that Cdh2 is a mEpiSC status-specific cadherin.

### Cdh2 is important for maintaining mEpiSCs in an undifferentiated state

**Figure 2:**
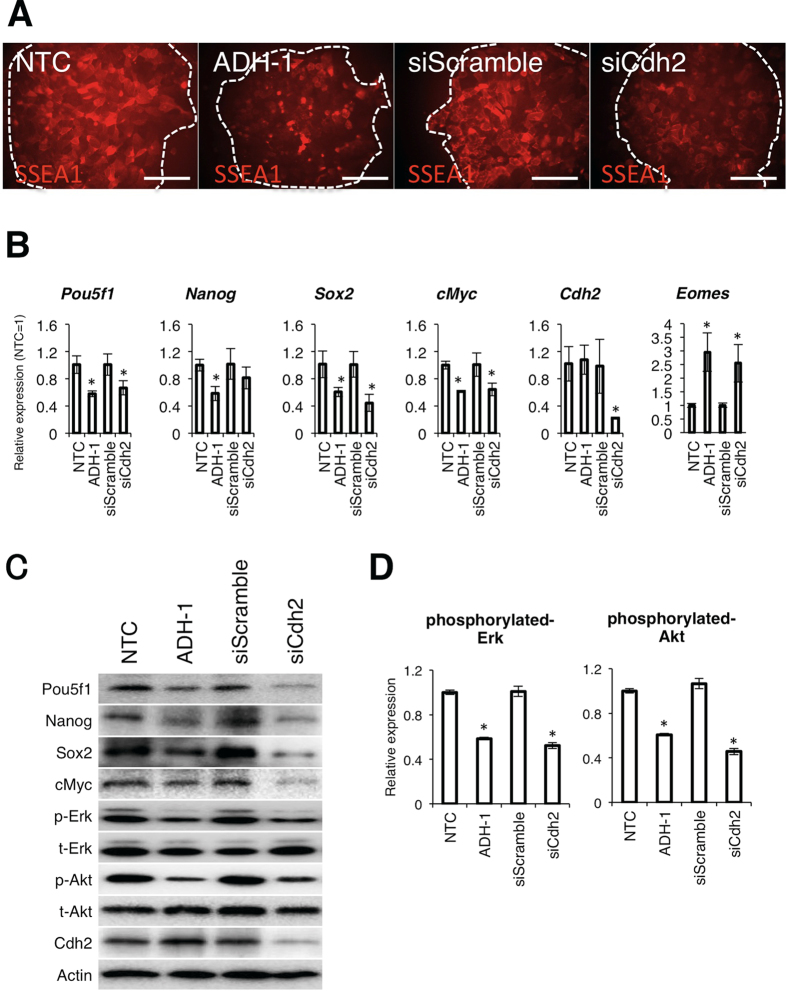
Cdh2 is important for maintenance of pluripotency in mEpiSCs. (**A**) SSEA-1 expression in control mEpiSCs (non-treated control: NTC) after treatment with ADH-1, control siRNA (siScramble) or Cdh2 siRNA (siCdh2). White dotted lines demarcate the colony regions. The scale bars are 100 μm. (**B**) qRT-PCR for the pluripotency-related genes *Pou5f1*, *Nanog*, *Sox2* and *cMyc*, the differentiation marker *Eomes* and *Cdh2* in non-treated mEpiSCs (non-treated control: NTC), ADH-1-treated mEpiSCs, control siRNA-treated mEpiSCs (siScramble) and Cdh2 siRNA-treated mEpiSCs (siCdh2). Data are normalized to the expression of *Lamina*. Bars represent the mean normalized values of triplicates. Asterisks indicate significant differences with *P* < 0.05. (**C**) WB analysis for Pou5f1, Nanog, Sox2, cMyc, phosphorylated-Erk (p-Erk), total-Erk (t-Erk), phosphorylated-Akt (p-Akt), total-Akt (t-Akt), Cdh2 and Actin in non-treated mEpiSCs (non-treated control: NTC), ADH-1-treated mEpiSCs, control siRNA-treated mEpiSCs (siScramble) and Cdh2 siRNA-treated mEpiSCs (siCdh2). (**D**) Densitometry quantification of WBs for phosphorylated-Erk and phosphorylated-Akt 24 hours after FGF2 treatment. Data are normalized to the expression levels of total-ErK and total-Akt. Bars represent the mean normalized values of triplicates. Asterisks indicate significant differences with *P* < 0.05.

To determine the relationship between Cdh2 and mEpiSCs pluripotency, we either disrupted Cdh2 function using ADH-1 (also known as Exherin), which is a selective inhibitor for Cdh2, or suppressed Cdh2 expression using specific siRNA to Cdh2 (siCdh2). ADH-1 and siCdh2 had no effect on colony size and viability (data not shown), but both treatments induced differentiation in mEpiSCs, as indicated by a decrease in SSEA-1, which is a pluripotent state-specific glycoprotein in mouse cells (Fig. 2A). We next analyzed the expression levels of other pluripotency-related genes in the ADH-1- and siCdh2-treated cells. A knockdown efficiency of ~80% occurred for siCdh2 compared with scrambled siRNA-transfected cells, as determined by qRT-PCR. The ADH-1 and the siCdh2 treatments both suppressed the expression of the *Pou5f1*, *Sox2* and *cMyc* genes at both the RNA and protein levels. Although *Nanog* mRNA expression did not decrease in siCdh2-treated cells, Nanog protein levels were lower in both ADH-1- and siCdh2-treated cells compared to non-treated cells. Expression of *Eomes*, which is an important transcription factor for neuronal and trophoblast differentiation, was upregulated by the ADH-1 and siCdh2 treatments (Fig. 2B). To elucidate the mechanism by which pluripotency-related genes are down-regulated by inhibition of Cdh2, we observed phosphorylation of Erk and Akt, both of which are important molecular mediators of pluripotency in primed-state stem cells^23^,^24^,^25^. WB analysis revealed that disruption of Cdh2 inhibited Erk and Akt phosphorylation (Fig. 2C,D).

### Overexpression of Cdh1 does not recover Cdh2 function

**Figure 3:**
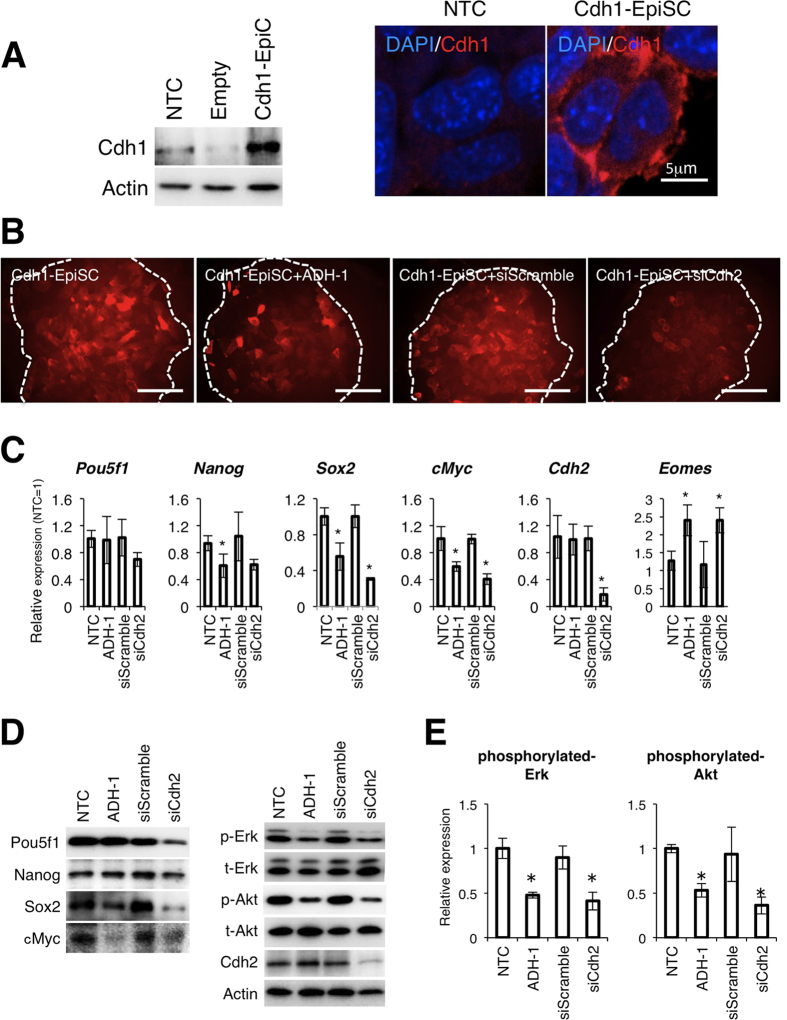
Overexpression of Cdh1 does not restore the function of Cdh2 in mEpiSCs. (**A**) The mEpiSC-line was transfected with an overexpression plasmid cording for mouse Cdh1 cDNA. Cdh1-expressing mEpiSCs (Cdh1-EpiSC) highly expressed the Cdh1 protein. NTC indicates control mEpiSCs and Empty indicates empty plasmid-transfected mEpiSCs. The right panel shows the localization of Cdh1. The scale bar is 5 μm. (**B**) SSEA-1 expression in Cdh1-EpiSCs after treatment with ADH-1 (Cdh1-EpiSC+ADH-1), control siRNA (Cdh1-EpiSC+siScramble) or Cdh2 siRNA (Cdh1-EpiSC+siCdh2). The white dotted lines demarcate the colony regions. Scale bars are 100 μm. (**C**) qRT-PCR for the pluripotency-related genes *Pou5f1*, *Nanog*, *Sox2* and *cMyc*, the differentiation marker *Eomes* and *Cdh2* in the non-treated Cdh1-EpiSCs (non-treated control: NTC), ADH-1-treated Cdh1-EpiSCs, control siRNA-treated Cdh1-EpiSCs (siScramble) and Cdh2 siRNA-treated Cdh1-EpiSCs (siCdh2). Data are normalized to the expression level of *Lamina*. Bars represent the mean normalized values of triplicates. Asterisks indicate significant differences with *P* < 0.05. (**D**) WB analysis for Pou5f1, Nanog, Sox2, cMyc, phosphorylated-Erk (p-Erk), total-Erk (t-Erk), phosphorylated-Akt (p-Akt), total-Akt (t-Akt), Cdh2 and Actin in non-treated Cdh1-EpiSCs (non-treated control: NTC), ADH-1-treated Cdh1-EpiSCs, control siRNA-treated Cdh1-EpiSCs (siScramble) and Cdh2 siRNA-treated Cdh1-EpiSCs (siCdh2). (**E**) Densitometry quantification of WBs for phosphorylated-Erk and phosphorylated-Akt. Data are normalized to the expression total-Erk and total-Akt. Bars represent the mean normalized values of triplicates. Asterisks indicate significant differences with *P* < 0.05.

To identify the specific function of Cdh2 and its significance in determining primed state pluripotency, we examined if Cdh1 could compensate for a loss of Cdh2 function in mEpiSCs. For this experiment, we produced a mEpiSC line (Cdh1-EpiSC) that stably expressed Cdh1 and maintained pluripotency in standard mEpiSC medium. Cdh1 localized to the surface of Cdh1-EpiSCs (Fig. 3A). Interestingly, even though Cdh1-EpiSCs strongly expressed cell surface Cdh1, some cells lost SSEA-1 expression after treatment with either ADH-1 or siCdh2 (Fig. 3B). To investigate this phenomenon in detail, we performed qRT-PCR and WB assays for pluripotency-related genes. ADH-1 suppressed the expression of *Nanog*, *Sox2* and *cMyc*, although Cdh1 expression rescued *Pou5f1*. Furthermore, consistent with the results from wild-type mEpiSCs, *Eomes* was upregulated by ADH-1 treatment. Treatment with siCdh2 had similar effects on Sox2, cMyc and *Eomes* expression (Fig. 3C). In addition, we found that siCdh2 treatment suppressed Erk and Akt phosphorylation in Cdh1-EpiSCs (Fig. 3D,E). These results suggest that Cdh2 is important for Erk and Akt phosphorylation and that suppression of Cdh2 decreases the expression of pluripotency-related genes.

### Cdh2 stabilizes FGFR1

**Figure 4:**
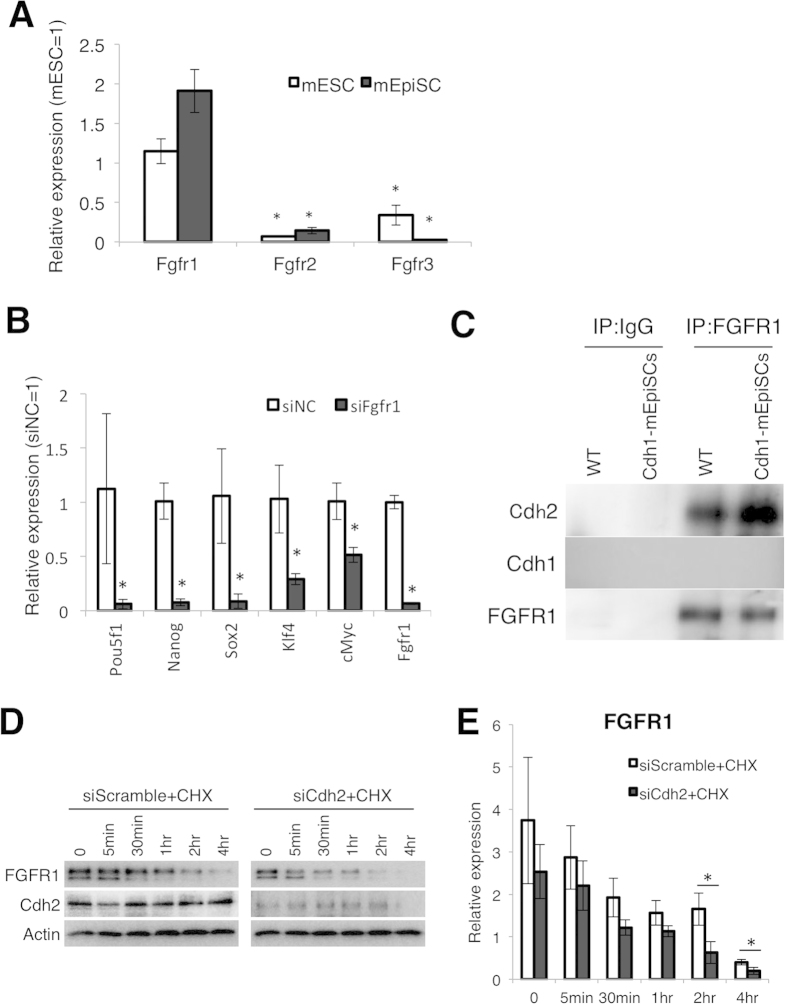
Cdh2 stabilizes FGFR1. (**A**) qRT-PCR for the FGFR family genes Fgfr1, Fgfr2 and Fgfr3 in mESCs and mEpiSCs. Bars represent the mean normalized values of triplicates. Asterisks indicate significant differences with *P* < 0.05. (**B**) qRT-PCR for pluripotency-related genes in the negative control siRNA-treated mEpiSCs (siNC) and the Fgfr1 siRNA-treated mEpiSCs (siFgfr1). Bars represent the mean normalized values of triplicates. Asterisks indicate significant differences with *P* < 0.05. (**C**) Co-immunoprecipitation (Co-IP) assay showing interaction between FGFR1 and Cdh1 in Cdh1-mEpiSCs and FGFR1 and Cdh2 in the wild-type mEpiSCs (WT). (**D**) WBs for FGFR1, Cdh2 and Actin in siRNA- and cycloheximide (CHX)-treated mEpiSCs 0, 5 min, 30 min, 1 hr, 2 hr and 4 hr after FGF2 stimulation. (**E**) Densitometry quantification of WBs for FGFR1, Cdh2 and Actin. Data are normalized to the expression of Actin. Bars represent the mean normalized values of triplicates. Asterisks indicate significant differences with *P* < 0.05.

Since Cdh2 disruption suppresses Akt and Erk phosphorylation, we focused our attention on the FGF signaling cascade, a pathway in which both Akt and Erk act as mediators. Since it is most likely for other molecules on the cell surface to interact with Cdh2, we hypothesized that Cdh2 may regulate FGF receptors (FGFRs). To test this hypothesis, we observed expression levels of three FGFRs in mEpiSCs. FGFR1 showed the highest expression in both mESCs and mEpiSCs. FGFR2 and FGFR3 expression levels were significantly lower than FGFR1 expression levels in mEpiSCs. The expression levels of FGFR2 and FGFR3 were 0.1-fold that of the FGFR1 level and were almost undetectable (Fig. 4A). Next, we examined the significance of FGFR1 in mEpiSCs pluripotency by introducing specific siRNA for Fgfr1 (siFgfr1). According to qRT-PCR, the siFgfr1 achieved a knockdown efficiency of 90% compared with scrambled siRNA. Treatment with siFgfr1 resulted in reduced expression of pluripotency-related genes (Fig. 4B). We next performed co-immunoprecipitation to confirm the interaction of Cdh2 with FGFR1 and to demonstrate that Cdh2 bound to FGFR1 in mEpiSCs (Fig. 4C). Furthermore, we determined if Cdh2 stabilized FGFR1 during FGF2 stimulation by analyzing FGFR1 degradation efficiency in cells treated with cycloheximide (CHX), which is an inhibitor of *de novo* protein synthesis. FGFR1 protein levels decreased in a time-dependent manner following CHX treatment, while Cdh2 expression did not change. The siCdh2-treatment accelerated FGFR1 protein degradation in these cells (Fig. 4D,E).

### Cdh2 does not affect the pluripotency of mESCs

**Figure 5:**
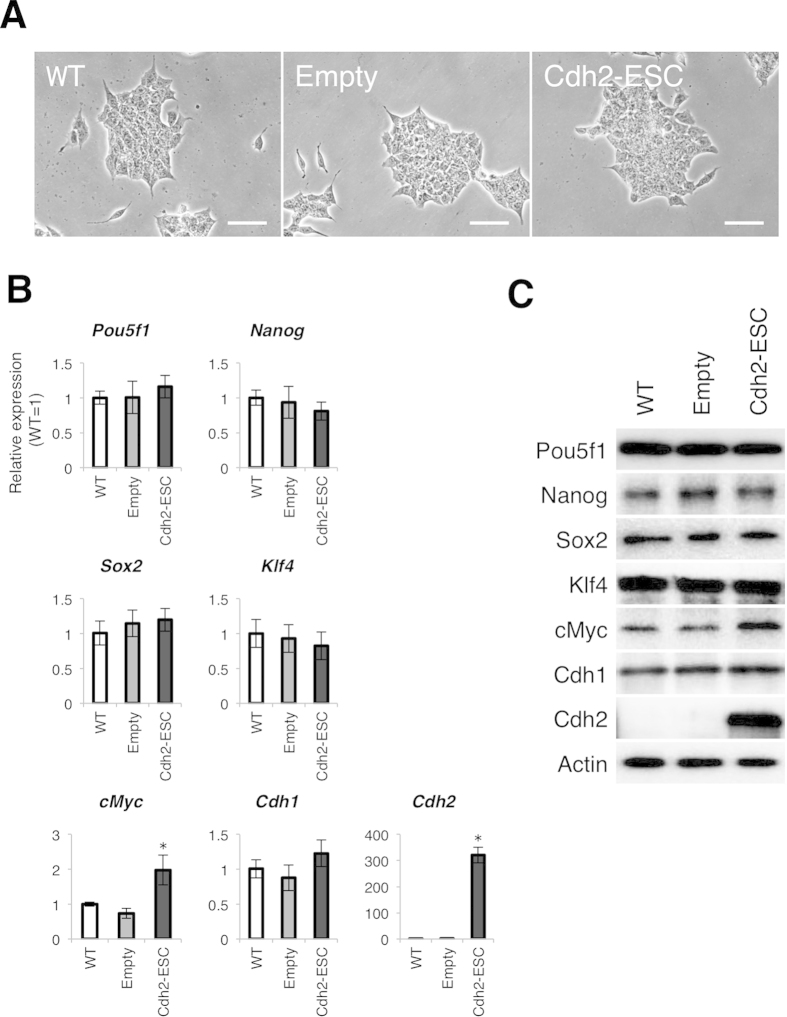
Cdh2 expression does not alter the pluripotent status of mESCs. (**A**) mESCs transfected with an overexpression plasmid cording for mouse Cdh2 cDN**A**. Morphological feature of wild-type mESCs (WT), empty plasmid-transfected mESCs (Empty) and Cdh2-overexpressed mESCs (Cdh2-ESC) are shown. The scale bars are 50 μm. (**B**) qRT-PCR for the pluripotency-related genes *Pou5f1*, *Nanog*, *Sox2*, *Klf4*, *cMyc*, as well as *Cdh1* and *Cdh2*, in the wild-type ESCs (WT), empty plasmid-transfected ESCs (empty) and *Cdh2*-overexpressed ESCs (Cdh2-ESC). Bars represent the mean normalized values of triplicates. Asterisks indicate significant differences with *P* < 0.05. (**C**) WBs for Pou5f1, Nanog, Sox2, Klf4, Cdh1, Cdh2 and Actin in wild-type mESCs (WT) and **C**dh2-overexpressed ESCs (Cdh2-ESC).

We also examined if Cdh2 affects the pluripotency of mESCs. We produced a Cdh2-overexpressing mESC-line (Cdh2-ESC) that formed typical dome-shaped mESC colonies (Fig. 5A) and proliferated at the same rate as the original mESC-line (data not shown). Our qRT-PCR and WB analyses revealed that *Pou5f1*, *Nanog* and *Sox2* expression did not change in Cdh2-ESCs (Fig. 5B,C). Interestingly, overexpression of Cdh2 upregulated cMyc mRNA and protein (Fig. 5B,C)

**Figure 6:**
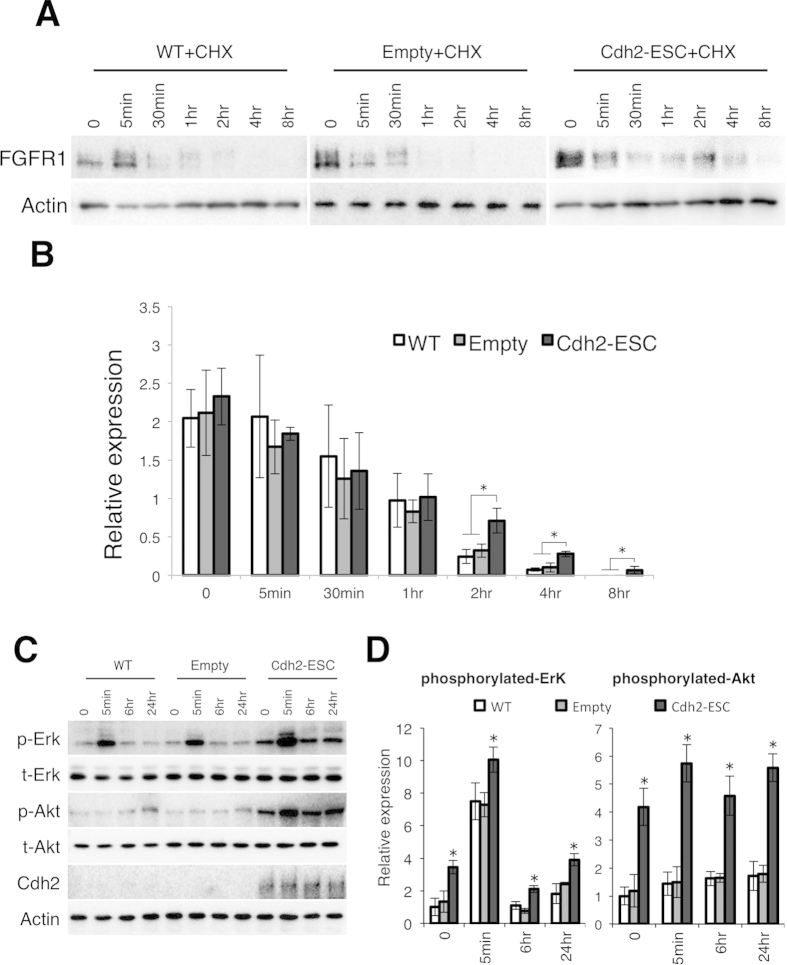
Expression of Cdh2 in mESCs alters FGFR1 stability along with the phosphorylation status of Erk and Akt under FGF2 stimulation. (**A**) WB analysis for FGFR1 in the CHX-treated wild-type mESCs (WT+CHX), CHX-treated empty plasmid-transfected mESCs (Empty+CHX) and CHX-treated Cdh2-ESCs (Cdh2-ESC+CHX) 0, 5 min, 30 min, 1 hr (1 hours), 2 hr (2 hours), 4 hr (4 hours) and 8 hr (8 hours) after FGF2 stimulation. (**B**) Densitometry quantification of WBs for FGFR1 and Actin. Data are normalized to the expression of Actin. Bars represent the mean normalized values of triplicates. Asterisks indicate significant differences with *P* < 0.05. (**C**) WB analysis for phosphorylated-Erk (p-Erk), total-Erk (t-Erk), phosphorylated-Akt (p-Akt), total-Akt (t-Akt), Cdh2 and Actin 0, 5 min, 6 hr and 24 hr after FGF2 stimulation. (**D**) Densitometry quantification of WBs for FGFR1 and Actin. Data are normalized to the expression total-Erk and -Akt. Bars represent the mean normalized values of triplicates. Asterisks indicate significant differences with *P* < 0.05.

To confirm that the overexpressed Cdh2 was functional, we observed the stability of FGFR1 in Cdh2-ESCs following treatment with CHX and FGF2. In wild-type mESCs, FGFR1 rapidly degraded. On the other hand, FGFR1 levels did not diminish in Cdh2-ESCs, even 8 hrs after FGF2 addition (Fig. 6A,B). These results clearly show that Cdh2 is important for FGFR1 stability. To verify Cdh2-mediated stabilization of FGFR1, we observed expression of phosphorylated Erk and Akt in Cdh2-ESCs after FGF2 stimulation. Phosphorylated Erk and Akt were significantly higher at all time-points in Cdh2-ESCs (Fig. 6C,D). These results support our hypothesis that Cdh2 is involved in the FGF-mediated signaling though interaction with FGFR1.

### Cdh2 supports FGF2-mediated differentiation in ESCs

**Figure 7:**
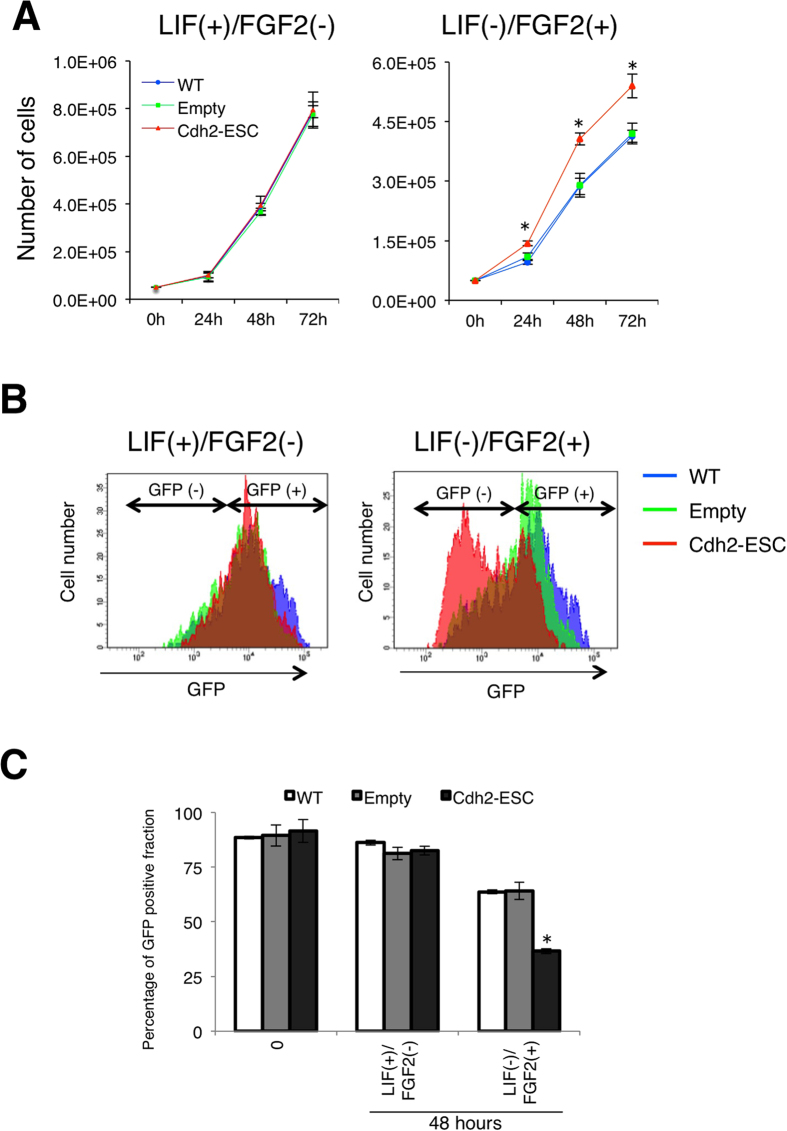
Cdh2 supports FGF2-induced proliferation and differentiation in mESCs. (**A**) Total cell numbers of wild-type mESCs (WT), empty plasmid-transfected mESCs (empty) and Cdh2-overexpressed mESCs (Cdh2-ESC) after 0, 24 h (24 hours), 48 h (48 hours) and 72 h (72 hours) of FGF2 stimulation. The left panel shows the growth curve for cells in LIF+/FGF2− medium and the right panel shows the growth curve for cells in LIF−/FGF2+ medium. (**B**,**C**) FACS analysis for Oct4 deltaPE-GFP-positive cells showing the naïve-state specific undifferentiated status of the cells. The left panel shows the GFP-positive Oct4 deltaPE-GFP mESCs from LIF+/FGF2− medium and right panel shows the GFP-positive Oct4 deltaPE-GFP mESCs from LIF−/FGF2+ medium after 48 hours. (**C**) The percentages of Oct4 deltaPE-GFP-positive cells for each culture condition were obtained by FACS analysis. Bars represent the mean values of triplicates. Asterisks indicate significant differences with *P* < 0.05.

FGF-Erk signaling induces differentiation of mESCs^26^. Thus, we examined if the overexpression of Cdh2 can accelerate FGF2-induced differentiation in mESCs. FGF2 is an important mitogenic cytokine for various types of cells including mEpiSCs^27^,^28^. We observed cell proliferation of wild-type and Cdh2-overexpressing ESCs under FGF2 stimulation 24 h, 48 h and 72 h after changing the medium. In LIF-supplemented medium, there was no difference between wild-type mESCs and Cdh2-ESCs (Fig. 7A). In contrast, there was a significant increase in cell number at each time-point in Cdh2-ESC compared with wild-type ESCs when they were cultured in FGF2-supplemented medium (Fig. 7A). This suggests that Cdh2 enhances the response of mESCs to FGF2. Next, we observed the efficiency of FGF2-induced differentiation using mESCs transfected with an Oct4 deltaPE-GFP reported plasmid, which contains a distal enhancer and the Oct4 promoter for naïve-state-specific GFP expression. In the LIF-supplemented medium, GFP-positive mESCs were present at the same ratio in wild-type ESCs as in Cdh2-ESCs (Fig. 7B). However, in the FGF2-supplemented culture medium, the number of GFP-positive cells significantly decreased in Cdh2-ESCs (Fig. 7B,C). These results confirm that Cdh2 contributes to FGF2-mediated differentiation of mESCs.

## Discussion

Cadherins are expressed in tissue- and cell-lineage-specific patterns that give rise to specific cell and tissue functions^29^,^30^,^31^,^32^,^33^,^34^. In this study, we found that mESCs express Cdh1 and Cdh3, while mEpiSCs express Cdh1 and Cdh2. The expression of Cdh1 mRNA was not consistent with its protein expression, suggesting posttranscriptional regulation of Cdh1, as reported previously^35^,^36^. Consistent with a previous study^37^, we observed that EpiSCs predominantly expressed Cdh2, suggesting a functional role of Cdh2 in regulating the pluripotency of mEpiSCs. Cdh2 knockdown decreased phosphorylated Erk and Akt, both of which are important for maintenance of pluripotency in primed-state stem cells^23^,^24^,^25^, and modified the expression of pluripotency-related genes. Interestingly, Cdh1 expression could not compensate for lost Cdh2 function, which prompted us to examine the molecular function of Cdh2 in pluripotency. Others have suggested that cadherins interact with various proteins, with partner protein binding varying by cadherin family member^38^,^39^. We focused our analysis on the phosphorylation status of Erk and Akt in siCdh2-treated mEpiSCs. FGF signaling regulates Erk and Akt phosphorylation in pluripotent stem cells^40^. We confirmed that suppression of FGFR1 attenuated pluripotency-related gene expression in mEpiSCs. This led us to hypothesize that Cdh2 contributes to a pluripotency-related network through its relationship with FGFR1, which we tested by observing protein-protein interactions between Cdh2 and FGFR1 in a co-IP assay. Suyama *et al*. reported that extracellular domain 4 of Cdh2 binds to Ig domains 1 and 2 of FGFR1, which inhibits internalization of FGFR1 and prevents FGFR1 degradation^41^. Hazan *et al*. reported that stabilizing Cdh2 on the cell membrane causes continuous tyrosine phosphorylation of FGFR1 by FGF2, with downstream activation of MAPK/Erk^42^. Consistent with these studies, we observed that Cdh2 stabilizes FGFR1 protein expression in CHX-treated mEpiSCs. Importantly, the observation that Cdh2 contributes to stabilization of FGFR1 and downstream Erk and Akt phosphorylation was reproducible in Cdh2-expressing mESCs. In Cdh2-ESCs, cMyc expression increased at both the mRNA and protein levels. This reasonable observation was supportive of our hypothesis, since it is well documented that cMyc expression is positively regulated by FGF2-Erk signaling^25^,^43^. Forced expression of Cdh2 enhanced FGF2-dependent cell proliferation in mESCs that were originally not responsive to FGF2. Furthermore, Cdh2-overexpression promoted FGF2-induced differentiation in mESCs. It has been reported that FGF signaling is detrimental to naïve-state pluripotency. However, our results suggest that Cdh2 is important for signal transduction of FGF2. Interestingly, Debiais *et al*. observed that FGF2 enhances the expression of Cdh2 in human calvaria osteoblasts through activation of protein kinase C and the Src-kinase pathway^44^. This suggests that there is a positive feedback-loop involving FGF and Cdh2.

On the other hand, the fact that our results indicate that Cdh2 contributes to pluripotency maintenance in mEpiSCs raises an important question about the similarity of EpiSCs to other primed-state stem cell types, such as human ESCs (hESCs) and rabbit ESCs (rbESCs). Until now, researchers have described primed-state pluripotent stem cells, including hESCs and rbESCs, according to their high Cdh1 expression to indicate their undifferentiated status, rather than by their Cdh2 expression level^45^,^46^. Furthermore, hESCs can lose Cdh1 and alternatively obtain Cdh2 expression during early differentiation processes^45^. This apparent contradiction may be accounted for if Cdh1 is not functional in the hESCs and rbESCs. Using mESCs, del Valle *et al*. demonstrated that Cdh1 can interact with β catenin and STAT3, and promote the LIF-dependent signaling cascades that are vital for naïve-pluripotency^21^. However, the LIF-dependent pathway does not function in hESCs and rbESCs. Thus, only low levels of Cdh2 may exist in hESCs and rbESCs, because it is dispensable for these cells. As we observed, mEpiSCs lacking Cdh2 maintain pluripotency-related gene expression, although the expression levels are attenuated compared with controls. Since Cdh2 supports FGFR1 stabilization, it may not be an essential co-factor/protective agent against the protein-degradation machinery. The importance of Cdh2 is thus likely to depend on the cellular status, for example, the type and level of cadherin that is expressed. In support of the importance of cellular differences between hESCs and mEpiSCs, Brons *et al*. reported significantly lower Rex1 expression in mEpiSCs compared to hESCs^15^. Rex1 is an important pluripotency-related gene in mESCs and hESCs^47^. There is also evidence that the primitive endodermal differentiation marker Gata6^48^,^49^ is not expressed in mESCs and hESCs, but is highly expressed in mEpiSCs^14^,^15^. Therefore, it is possible that mEpiSCs display a more differentiated state than other primed-state ESCs. These differences may result in altered cadherin expression patterns.

In this study, we only focused on Cdh2 function and its interaction with FGFR1. However, we cannot ignore other possible functions of cadherins. Recent research has elucidated direct interactions between cadherins and transcription factors that influence gene expression. For example, the intercellular-domain of Cdh2 from the presenilin1/gamma secretase complex binds to a transcriptional co-activator CREB-binding protein (CBP) and induces proteasome-mediated CBP degradation^50^. These reactions can directly suppress CBP/CREB-mediated transcription, leading to Cdh1 modulation of cell growth and epithelial-mesenchymal transition through interaction of β-catenin transcriptional activity in cancer cells and epithelial cells^51^,^52^. Furthermore, Cdh13 suppresses activation of c-Jun/JNK and induces G2/M cell cycle arrest in hepatocellular carcinoma cells^53^. Recently, Sancisi *et al*. reported that Cdh6 interacts with Runx2 in thyroid tumor cells^54^. These results suggest functional diversity in the cadherin proteins and the possibility that cadherins contribute to significant changes in cell fate over long periods. Further research on cadherin expression patterns in various stem cells and a comparison of cadherin expression levels according to differentiation status will be essential for a complete understanding of stem cell pluripotency.

## Materials and Methods

### Animal use and care

This project and all related experimental protocols were approved by the Kinki University Animal Experimental Ethics Committee for Laboratory Experimentation (project number: KAME-26-043) and were carried out in accordance with the approved guidelines.

### mESC and mEpiSC Cell Culture

Mouse wild-type ESCs (C57BL/6J), Oct4 deltaPE-GFP expressing ESCs and wild-type EpiSCs (C57BL/6J) were produced as described previously^22^. The mESCs were cultured on gelatin-coated dishes in mESC medium, which consisted of Knockout DMEM with 1× MEM nonessential amino acids, 0.1 mM 2-mercaptoethanol, 200 mM L-glutamine and 20% Knockout Serum Replacement (all purchased from Life Technologies Inc., CA, USA), supplemented with 1000 units/ml ESGRO (Millipore, MA, USA). The mEpiSCs were cultured in mEpiSC medium, which consisted of Knockout DMEM with 1× MEM nonessential amino acids, 0.1 mM 2-mercaptoethanol, 200 mM L-glutamine and 20% Knockout Serum Replacement, supplemented with 5 μg/ml FGF2 (Wako Pure Chemical Industries, Ltd., Osaka, Japan). To passage the cells, 10 μM Y-27632 (Wako) was added to the medium. ADH-1 (Exherin™; MedKoo Biosciences, Inc., NC, USA), which is a competitive inhibitor of N-cadherin^55^, was dissolved in PBS (−) and added to the medium at a concentration of 0.2 mg/ml.

### Production of Stable Cdh1-Expressing mEpiSCs and Cdh2-Expressing mESCs

To create the pCAG-Ncadherin-IRES-Puro plasmid, the Ncadherin ORF sequences from the Ncadherin-GFP plasmid, which was purchased from Addgene (number #18870), were cloned into the EcoRI site of the pCAG-IRES-Puro plasmid, which was kindly provided by Dr. Hirofumi Suemori. The pCAG-Ecadherin-IRES-Puro plasmid was kindly provided by Dr. Hitoshi Niwa. The mESCs and mEpiSCs were dissociated to single cells by Trypsin-EDTA treatment and resuspended in PBS (−) with either the linearized pCAG-Ncadherin-IRES-Puro or pCAG-Ecadherin-IRES-Puro vector. These cell suspensions were transferred to a Gene Pulser cuvette (0.4-cm gap), electroporated with a Gene Pulser II (Bio-Rad Laboratories, CA, USA) at 200 V and 900 μF and plated on Matrigel (Corning Japan, Tokyo, Japan) -coated dishes. After 24 hours, 0.4 μg/ml Puromycin (Life Technologies) was added to the culture medium for drug selection for 48 hr.

### Suppression of Cdh1, Cdh2 and Fgfr1 mRNA by siRNA treatment

Cdh1, Cdh2 and Fgfr1 siRNAs (5 pmol) were transfected into the cells using Lipofectamine RNAiMAX Reagent (Life Technologies). After 48 hr of transfection, the cells were collected for qRT-PCR and WB based analyses. The siRNA sequences are given in Supplementary Table S2.

### Immunofluorescent Staining

Cells were fixed in Mildform^®^ (Wako) for 30 min and permeabilized in 0.2% Triton-X (Sigma-Aldrich Corporation, MO, USA) in PBS (−) for 10 min. The fixed samples were blocked by incubation in 10% Block Ace (Dainippon Sumitomo Pharma, Osaka, Japan) in PBS (−) for 1 h and incubated with anti-Cdh1 (Santa Cruz Biotechnology, Inc., CA, USA) and anti-SSEA1 (Santa Cruz Biotechnology) primary antibodies diluted in PBS (−) containing 10% Block Ace overnight at 4 °C. The samples were then washed twice with PBS (−) and reacted with TexasRed-conjugated anti-rabbit IgG antibody or PE-conjugated anti-mouse IgM antibody (Santa Cruz Biotechnology) diluted in PBS (−) containing 10% Block Ace for 1 hour in the dark. Samples were counter-stained with DAPI (Vector Laboratories Ltd., Peterborough, England) before microscopic observation. Images were acquired using a fluorescence microscope (Keyence Corporation, Osaka, Japan).

### Quantitative RT-PCR (qRT-PCR) analysis

Total RNA was collected using a TRI Reagent® (Molecular Research Center, Inc., OH, USA) and a PrimeScript® RT Master Mix Kit (TAKARA Bio Inc., Shiga, Japan). Quantitative RT-PCR for total cDNA was performed using Perfect real-time SYBR green II (Takara). PCR amplifications were performed using a Thermal Cycler Dice® Real Time System Single at 95 °C for 20s followed by 40 cycles of 95 °C for 5s and 60 °C for 30s. To quantify the relative expression of each gene, the Ct (threshold cycle) values were normalized by Lamina (ΔCt = Ct_target_ − Ct_Lamina_) and compared with a calibrator using the “ΔΔCt method” (ΔΔCt = ΔCt_sample_ − ΔCt_calibrator_). All values are means ± SD of 3 experiments. Statistical significance was evaluated with JMP software version 10.0.0 (SAS Institute, Cary, NC, USA) using either Student’s t-test or the Tukey-Kramer HSD test. The primer sequences are given in Supplementary Table S1.

### Western Blot (WB) Analysis

Samples were homogenized in SDS buffer (4% SDS, 125 mM Tris–glycine, 10% -mercaptoethanol, 2% bromophenol blue in 30% glycerol) and centrifuged at 10,000 rcf for 10 min at 4 °C to remove debris. Aliquots were subjected to polyacrylamide gel electrophoresis in the presence of SDS (SDS/PAGE) followed by electrotransfer onto a PVDF membrane (Hybond-P; GE Healthcare Japan, Tokyo, Japan). The blotted membranes were blocked overnight with Block Ace (Dainippon Sumitomo Pharma) and then probed with primary antibody overnight at 4 °C. Detection was performed with horseradish peroxidase (HRP)-conjugated secondary antibodies (all antibody were purchased from Santa Cruz Biotechnology) and either the ECL prime Western blotting detection system (GE Healthcare Japan) or Immunostar^®^ LD (Wako). The lumino-labeled membranes were analyzed using a CCD-based chemiluminescent analyzer (Amersham™ Imager 600, GE Healthcare Japan). To assess the expression level, relative band intensities were estimated using ImageQuant™ TL (GE Healthcare Japan). All values are represented as means ± SD of 3 experiments. Statistical significance was evaluated by Student’s t-test using JMP software. Antibodies are descried in Supplementary Table S3.

### Co-Immunoprecipitation (Co-IP)

Samples were lysed in IP extraction buffer (25 mM Tris-HCl pH7.5, 100 m NaCl, 0.5% Triton X-100) for 30 min on ice. The samples were then centrifuged to collect the supernatant. An anti-FGFR1 antibody (Cell Signaling Technology, Inc., MA, USA) with Dynabeads Protein G (Veritas Corporation., Tokyo, Japan) in 0.02% tween TBS buffer was used for the Co-IP reaction. After Co-IP, proteins were eluted in SDS buffer for WB analysis. The antibodies are described in Supplementary Table S3.

### Flow cytometry (FACS) analysis

Wild-type- and Cdh2- ESCs were dissociated into single cells by Trypsin-EDTA after 48 hours of treatment, washed twice and diluted in the FACS buffer (2% FBS and 10 mM HEPES in DMEM). The Oct4 deltaPE-GFP positive cell fractions were analyzed by flow cytometry with a FACS Caliber cytometer (Becton Dickinson, Franklin Lakes, NJ). All values are means ± SD of 3 experiments. Statistical significance was evaluated by Student’s t-test using JMP software.

### Cell counts

A total of 5 × 10^4^ wild-type, empty- or Cdh2- ESCs were plated per well of a 24 well plate. After 24, 48 and 72 hour, the cells were trypsinized into single cell suspensions and automatically counted using a TC20™ Automated Cell Counter (Bio-Rad Laboratories).

### Inhibition of Protein Synthesis by Cycloheximide

We observed the expression of various cadherin types and the degradation of FGFR1 protein by activation of FGFR1/Mek-Erk following FGF2 stimulation. After FGF2 stimulation and cycloheximide treatment (final concentration is 25 μm) for 0, 5 min, 30 min, 1 hr, 2 hr, 4 hr and 8 hr, all samples were eluted in 2× SDS sample buffer and analyzed by Western blot using anti-Cdh2, anti-FGFR1 and anti-Actin antibodies (detailed information in Supplementary Table S3).

## Additional Information

**How to cite this article**: Takehara, T. *et al*. Cdh2 stabilizes FGFR1 and contributes to primed-state pluripotency in mouse epiblast stem cells. *Sci. Rep*. **5**, 14722; doi: 10.1038/srep14722 (2015).

## Supplementary Material

Supplementary Information

## References

[b1] Evans M. J. & Kaufman M. H. Establishment in culture of pluripotential cells from mouse embryos. Nature 292, 154–156 (1981). 7242681 10.1038/292154a0

[b2] Martin G. R. Isolation of a pluripotent cell line from early mouse embryos cultured in medium conditioned by teratocarcinoma stem cells. Proc Natl Acad Sci USA 78, 7634–7638 (1981). 6950406 10.1073/pnas.78.12.7634 PMC349323

[b3] Buehr M. . Capture of authentic embryonic stem cells from rat blastocysts. Cell 135, 1287–1298, 10.1016/j.cell.2008.12.007 (2008). 19109897

[b4] Suemori H. . Establishment of embryonic stem cell lines from cynomolgus monkey blastocysts produced by IVF or ICSI. Dev Dyn 222, 273–279, 10.1002/dvdy.1191 (2001). 11668604

[b5] Thomson J. A. . Embryonic stem cell lines derived from human blastocysts. Science 282, 1145–1147 (1998). 9804556 10.1126/science.282.5391.1145

[b6] Nichols J. & Smith A. Naive and primed pluripotent states. Cell Stem Cell 4, 487–492, 10.1016/j.stem.2009.05.015 (2009). 19497275

[b7] Boeuf H., Hauss C., Graeve F. D., Baran N. & Kedinger C. Leukemia inhibitory factor-dependent transcriptional activation in embryonic stem cells. J Cell Biol 138, 1207–1217 (1997). 9298977 10.1083/jcb.138.6.1207 PMC2132559

[b8] Buecker C. . A murine ESC-like state facilitates transgenesis and homologous recombination in human pluripotent stem cells. Cell Stem Cell 6, 535–546, 10.1016/j.stem.2010.05.003 (2010). 20569691 PMC3162213

[b9] Gu Q. . Rapid conversion of human ESCs into mouse ESC-like pluripotent state by optimizing culture conditions. Protein Cell 3, 71–79, 10.1007/s13238-012-2007-8 (2012). 22271597 PMC4875221

[b10] Hanna J. . Human embryonic stem cells with biological and epigenetic characteristics similar to those of mouse ESCs. Proc Natl Acad Sci USA 107, 9222–9227, 10.1073/pnas.1004584107 (2010). 20442331 PMC2889088

[b11] Gafni O. . Derivation of novel human ground state naive pluripotent stem cells. Nature 504, 282–286, 10.1038/nature12745 (2013). 24172903

[b12] Ware C. B. . Derivation of naive human embryonic stem cells. Proc Natl Acad Sci USA 111, 4484–4489, 10.1073/pnas.1319738111 (2014). 24623855 PMC3970494

[b13] Theunissen T. W. . Systematic identification of culture conditions for induction and maintenance of naive human pluripotency. Cell Stem Cell 15, 471–487, 10.1016/j.stem.2014.07.002 (2014). 25090446 PMC4184977

[b14] Tesar P. J. . New cell lines from mouse epiblast share defining features with human embryonic stem cells. Nature 448, 196–199, 10.1038/nature05972 (2007). 17597760

[b15] Brons I. G. . Derivation of pluripotent epiblast stem cells from mammalian embryos. Nature 448, 191–195, 10.1038/nature05950 (2007). 17597762

[b16] Pieters T. & van Roy F. Role of cell-cell adhesion complexes in embryonic stem cell biology. J Cell Sci 127, 2603–2613, 10.1242/jcs.146720 (2014). 24931943

[b17] Spencer H. L. . E-cadherin inhibits cell surface localization of the pro-migratory 5T4 oncofetal antigen in mouse embryonic stem cells. Mol Biol Cell 18, 2838–2851, 10.1091/mbc.E06-09-0875 (2007). 17507657 PMC1949355

[b18] Takeichi M. Cadherins: a molecular family important in selective cell-cell adhesion. Annual review of biochemistry 59, 237–252, 10.1146/annurev.bi.59.070190.001321 (1990). 2197976

[b19] Drees F., Pokutta S., Yamada S., Nelson W. J. & Weis W. I. Alpha-catenin is a molecular switch that binds E-cadherin-beta-catenin and regulates actin-filament assembly. Cell 123, 903–915, 10.1016/j.cell.2005.09.021 (2005). 16325583 PMC3369825

[b20] Pey R., Vial C., Schatten G. & Hafner M. Increase of intracellular Ca2+ and relocation of E-cadherin during experimental decompaction of mouse embryos. Proc Natl Acad Sci USA 95, 12977–12982 (1998). 9789026 10.1073/pnas.95.22.12977 PMC23677

[b21] del Valle I. . E-cadherin is required for the proper activation of the Lifr/Gp130 signaling pathway in mouse embryonic stem cells. Development 140, 1684–1692, 10.1242/dev.088690 (2013). 23487312

[b22] Takehara T., Teramura T., Onodera Y., Hamanishi C. & Fukuda K. Reduced oxygen concentration enhances conversion of embryonic stem cells to epiblast stem cells. Stem Cells Dev 21, 1239–1249, 10.1089/scd.2011.0322 (2012). 21861689

[b23] Lanner F. & Rossant J. The role of FGF/Erk signaling in pluripotent cells. Development 137, 3351–3360, 10.1242/dev.050146 (2010). 20876656

[b24] Hassani S. N., Totonchi M., Gourabi H., Scholer H. R. & Baharvand H. Signaling roadmap modulating naive and primed pluripotency. Stem Cells Dev 23, 193–208, 10.1089/scd.2013.0368 (2014). 24147644

[b25] Armstrong L. . The role of PI3K/AKT, MAPK/ERK and NFkappabeta signalling in the maintenance of human embryonic stem cell pluripotency and viability highlighted by transcriptional profiling and functional analysis. Hum Mol Genet 15, 1894–1913, 10.1093/hmg/ddl112 (2006). 16644866

[b26] Kunath T. . FGF stimulation of the Erk1/2 signalling cascade triggers transition of pluripotent embryonic stem cells from self-renewal to lineage commitment. Development 134, 2895–2902, 10.1242/dev.02880 (2007). 17660198

[b27] Greber B. . Conserved and divergent roles of FGF signaling in mouse epiblast stem cells and human embryonic stem cells. Cell Stem Cell 6, 215–226, 10.1016/j.stem.2010.01.003 (2010). 20207225

[b28] Adepoju A., Micali N., Ogawa K., Hoeppner D. J. & McKay R. D. FGF2 and insulin signaling converge to regulate cyclin D expression in multipotent neural stem cells. Stem Cells 32, 770–778, 10.1002/stem.1575 (2014). 24155149

[b29] Halbleib J. M. & Nelson W. J. Cadherins in development: cell adhesion, sorting, and tissue morphogenesis. Genes Dev 20, 3199–3214, 10.1101/gad.1486806 (2006). 17158740

[b30] Peinado H., Portillo F. & Cano A. Transcriptional regulation of cadherins during development and carcinogenesis. Int J Dev Biol 48, 365–375, 10.1387/ijdb.041794hp (2004). 15349812

[b31] Wheelock M. J., Shintani Y., Maeda M., Fukumoto Y. & Johnson K. R. Cadherin switching. J Cell Sci 121, 727–735, 10.1242/jcs.000455 (2008). 18322269

[b32] Berx G. & van Roy F. Involvement of members of the cadherin superfamily in cancer. Cold Spring Harb Perspect Biol 1, a003129, 10.1101/cshperspect.a003129 (2009). 20457567 PMC2882122

[b33] Shintani Y., Hollingsworth M. A., Wheelock M. J. & Johnson K. R. Collagen I promotes metastasis in pancreatic cancer by activating c-Jun NH(2)-terminal kinase 1 and up-regulating N-cadherin expression. Cancer Res 66, 11745–11753, 10.1158/0008-5472.CAN-06-2322 (2006). 17178870

[b34] Li G., Satyamoorthy K. & Herlyn M. N-cadherin-mediated intercellular interactions promote survival and migration of melanoma cells. Cancer Res 61, 3819–3825 (2001). 11325858

[b35] Palacios F., Tushir J. S., Fujita Y. & D’Souza-Schorey C. Lysosomal targeting of E-cadherin: a unique mechanism for the down-regulation of cell-cell adhesion during epithelial to mesenchymal transitions. Mol Cell Biol 25, 389–402, 10.1128/MCB.25.1.389-402.2005 (2005). 15601859 PMC538771

[b36] Song S. . Pou5f1-dependent EGF expression controls E-cadherin endocytosis, cell adhesion, and zebrafish epiboly movements. Dev Cell 24, 486–501, 10.1016/j.devcel.2013.01.016 (2013). 23484854 PMC3598594

[b37] Bao S. . Epigenetic reversion of post-implantation epiblast to pluripotent embryonic stem cells. Nature 461, 1292–1295, 10.1038/nature08534 (2009). 19816418 PMC3863718

[b38] Pece S. & Gutkind J. S. Signaling from E-cadherins to the MAPK pathway by the recruitment and activation of epidermal growth factor receptors upon cell-cell contact formation. J Biol Chem 275, 41227–41233, 10.1074/jbc.M006578200 (2000). 10969083

[b39] Wheelock M. J. & Johnson K. R. Cadherin-mediated cellular signaling. Curr Opin Cell Biol 15, 509–514 (2003). 14519384 10.1016/s0955-0674(03)00101-7

[b40] Li J. . MEK/ERK signaling contributes to the maintenance of human embryonic stem cell self-renewal. Differentiation 75, 299–307, 10.1111/j.1432-0436.2006.00143.x (2007). 17286604

[b41] Suyama K., Shapiro I., Guttman M. & Hazan R. B. A signaling pathway leading to metastasis is controlled by N-cadherin and the FGF receptor. Cancer Cell 2, 301–314 (2002). 12398894 10.1016/s1535-6108(02)00150-2

[b42] Hazan R. B., Phillips G. R., Qiao R. F., Norton L. & Aaronson S. A. Exogenous expression of N-cadherin in breast cancer cells induces cell migration, invasion, and metastasis. J Cell Biol 148, 779–790 (2000). 10684258 10.1083/jcb.148.4.779 PMC2169367

[b43] Lepique A. P. . c-Myc protein is stabilized by fibroblast growth factor 2 and destabilized by ACTH to control cell cycle in mouse Y1 adrenocortical cells. J Mol Endocrinol 33, 623–638, 10.1677/jme.1.01485 (2004). 15591023

[b44] Debiais F. . Fibroblast growth factor-2 (FGF-2) increases N-cadherin expression through protein kinase C and Src-kinase pathways in human calvaria osteoblasts. Journal of cellular biochemistry 81, 68–81 (2001). 11180398 10.1002/1097-4644(20010401)81:1<68::aid-jcb1024>3.0.co;2-s

[b45] Eastham A. M. . Epithelial-mesenchymal transition events during human embryonic stem cell differentiation. Cancer Res 67, 11254–11262, 10.1158/0008-5472.CAN-07-2253 (2007). 18056451

[b46] Teramura T. . Induction of functional mesenchymal stem cells from rabbit embryonic stem cells by exposure to severe hypoxic conditions. Cell Transplant 22, 309–329, 10.3727/096368912X653291 (2013). 22943955

[b47] Ludwig T. E. . Derivation of human embryonic stem cells in defined conditions. Nat Biotechnol 24, 185–187, 10.1038/nbt1177 (2006). 16388305

[b48] Rong L. . GATA-6 promotes cell survival by up-regulating BMP-2 expression during embryonic stem cell differentiation. Mol Biol Cell 23, 3754–3763, 10.1091/mbc.E12-04-0313 (2012). 22855527 PMC3442421

[b49] Lee J. H. . Spontaneously differentiated GATA6-positive human embryonic stem cells represent an important cellular step in human embryonic development; they are not just an artifact of *in vitro* culture. Stem Cells Dev 22, 2706–2713, 10.1089/scd.2013.0083 (2013). 23746070 PMC3787399

[b50] Marambaud P. . A CBP binding transcriptional repressor produced by the PS1/epsilon-cleavage of N-cadherin is inhibited by PS1 FAD mutations. Cell 114, 635–645 (2003). 13678586 10.1016/j.cell.2003.08.008

[b51] Conacci-Sorrell M. . Autoregulation of E-cadherin expression by cadherin-cadherin interactions: the roles of beta-catenin signaling, Slug, and MAPK. J Cell Biol 163, 847–857, 10.1083/jcb.200308162 (2003). 14623871 PMC2173691

[b52] Stockinger A., Eger A., Wolf J., Beug H. & Foisner R. E-cadherin regulates cell growth by modulating proliferation-dependent beta-catenin transcriptional activity. J Cell Biol 154, 1185–1196, 10.1083/jcb.200104036 (2001). 11564756 PMC2150811

[b53] Chan D. W., Lee J. M., Chan P. C. & Ng I. O. Genetic and epigenetic inactivation of T-cadherin in human hepat{Chan, 2008 #11}ocellular carcinoma cells. Int J Cancer 123, 1043–1052, 10.1002/ijc.23634 (2008). 18553387

[b54] Sancisi V. . Cadherin 6 is a new RUNX2 target in TGF-β signalling pathway. PLoS One 8, e75489, 10.1371/journal.pone.0075489 (2013). 24069422 PMC3772092

[b55] Shintani Y. . ADH-1 suppresses N-cadherin-dependent pancreatic cancer progression. Int J Cancer 122, 71–77, 10.1002/ijc.23027 (2008). 17721921

