# Supplementary material for: Cdh2 stabilizes FGFR1 and contributes to primed-state pluripotency in mouse epiblast stem cells
Source: Sci Rep. 2015 Sep 30;5:14722. doi: 10.1038/srep14722 (PMC4588589; doi:10.1038/srep14722)
Supplement: Supplementary Information [file srep14722-s1.pdf]

Supplementary Online Material for

Cdh2 stabilizes FGFR1 and contributes to primed-state pluripotency in mouse epiblast stem cells

Toshiyuki Takehara, Takeshi Teramura, Yuta Onodera, John Frampton and Kanji Fukuda

Correspondence should be addressed to Takeshi Teramura (teramura@med.kindai.ac.jp)

Supplementary Table S1.

Primer sequence used for qRT-PCR analysis.

Primer Name	Primer Sequence 5'-3'	
Lamina (Lamin A)	Forward	AGCACTGCTCTCAGTGAGAAG
	Reverse	TCCTGAAGCTGCTTCTTAGC
Pou5f1	Forward	GATGGTGGTCTGGCTGAACA
	Reverse	GCAGAAGAGGATCACCTTGG
Nanog	Forward	ACCAGTGGTTGAAGACTAGCAAT
	Reverse	CTGCAATGGATGCTGGGATA
Sox2	Forward	CTGTTTTTTCATCCCAATTGCAC
	Reverse	AGATCTGGCGGAGAATAGTTG
Klf4	Forward	AGTTCTCATCTCAAGGCACAC
	Reverse	TCACAGTGGTAAGGTTTCTCG
cMyc	Forward	GTCTTTCCCTACCCGCTCA
	Reverse	TGGAATCGGACGAGGTACAG
Eomes	Forward	CACCCAGCTAAAGATCGACCA
	Reverse	TTTCTGAAGCCGTGTACATGGA
Cdh1 (E-cadherin)	Forward	TCTGTCGCCACTTTGAATC
	Reverse	ATGTCCTGGGCAGAGTGAG
Cdh2 (N-cadherin)	Forward	TGACTGAGGAGCCTATGAAG
	Reverse	TTGTAGAGCTCCACTGTGC
Cdh3 (P-cadherin)	Forward	TATGCCACTGGACAGGG
	Reverse	ATTCTCAGATACAGCGTGGC
Cdh4 (R-cadherin)	Forward	TTCCCTCAGCAGCTTGTC
	Reverse	TGATGCTGTAGCGGATG
Cdh5 (VE-cadherin)	Forward	TGTGCCAGAGATGTCAGC
	Reverse	TACAAGACAGTGGCGTGG
Cdh12 (N-cadherin type2)	Forward	TGCTGGAAGAGTACATGG
	Reverse	AGTGCCCTCTCCTTTATCC
Cdh13 (H-cadherin)	Forward	TGGACAGAGAAACGATCG
	Reverse	TCTGTTGTCGTTCTGGTC
Cdh15 (M-cadherin)	Forward	AAGGTGTGCTGTCCGTG
	Reverse	TGAACCCACACGCTGAC
Fgfr1	Forward	TGGCTTAGCTCGAGACATTC
	Reverse	ATCCACTTCACAGGCAGC
Fgfr2	Forward	TGGCAGTGAAGATGTTGAAAG
	Reverse	ATCATCTTCATCATCTCCATCTCTG
Fgfr3	Forward	TGAGCTGCCTGCTGACCC
	Reverse	CCAAAGCAGCCTTCTCCAA

Supplementary Table 2.

Sequences of siRNAs used for knockdown experiments in the present study.

siRNA	Sequence 5'–3'	
Cadherin2 #1	SIGMA	CUGAGUUUCUGCACCAGGUTT<br>ACCUGGUGCAGAAACUCAGTT
Cadherin2 #2	SIGMA	GAGUUUACUGCCAUGACUUTT<br>AAGUCAUGGCAGUAAACUCTT
Cadherin2 #3	SIGMA	GGAUGUUUGUCCUUACUGUTT<br>ACAGUAAGGACAAACAUCCTT
Scramble (negative control)	SIGMA	UACUAUUCGACACGCGAAGTT<br>CUUCGCGUGUCGAAUAGUATT
Fgfr1	Life Technologies	s66025
Silencer® Select Negative Control #1 siRNA	Life Technologies	4390843

Supplementary Table S3.

The antibodies used for western blot (WB), immunofluorescence (IF) and co-immunoprecipitation (Co-IP) in the present study.

Primary antibody				
	Antibody	Company	Dilution	kDa
WB/ IF	OCT-3/4 (sc-9081)	Santa Cruz Biotechnology	1/5,000 in Immuno-enhancer	45
	NANOG (14-5761-80)	eBioscience	1/1,000 in Immuno-enhancer	38
	SOX2 (sc-17320)	Santa Cruz Biotechnology	1/500 in Immuno-enhancer	38
	GKLF (sc-20691)	Santa Cruz Biotechnology	1/5,000 in Immuno-enhancer	55
	Cadherin1 (E-cadherin) (sc-7870)	Santa Cruz Biotechnology	1/1,000 in Immuno-enhancer	120
	Cadherin2 (N-cadherin) (sc-7939)	Santa Cruz Biotechnology	1/1,000 in Immuno-enhancer	130
	GAPDH (3C2 / H00002579-A01)	Abnova	1/10,000 in 10% Block Ace - TBST	38
	Total-ERK1/2 (#4695)	Cell Signaling Technology	1/5,000 in Immuno-enhancer	42 / 44
	phopshorylation-ERK1/2 (#9101)	Cell Signaling Technology	1/3,000 in Immuno-enhancer	42 / 44
	Total-AKT (#9272)	Cell Signaling Technology	1/5,000 in Immuno-enhancer	60
	phopshorylation-AKT (#9271)	Cell Signaling Technology	1/1,000 in Immuno-enhancer	60
	FGFR1 (#9740)	Cell Signaling Technology	1/3,000 in Immuno-enhancer	120 / 145
	FGFR1 (19B2 / #05-149)	MERCK MILLIPORE	1/5,000 in Immuno-enhancer	-120
	SSEA1 (sc-21702)	Santa Cruz Biotechnology	1/200 in 10% Block Ace - PBS(-)	-
	ACTIN (sc-1616)	Santa Cruz Biotechnology	1/10,000 in 10% Block Ace - TBST	43
Co-IP	Normal rabbit IgG (sc-2027)	Santa Cruz Biotechnology	1/200 in Co-IP buffer	
	FGFR1 (#9740)	Cell Signaling Technology	1/200 in Co-IP buffer	
Secondary				
WB/ IF	Donkey anti goat IgG-HRP (sc-2020)	Santa Cruz Biotechnology	1/50,000 in Immuno-enhancer 1/50,000 in 10% Block Ace - TBST	
	Goat anti rat IgG-HRP (sc-2006)	Santa Cruz Biotechnology	1/50,000 in Immuno-enhancer	
	Goat anti rabbit IgG-HRP (sc-2004)	Santa Cruz Biotechnology	1/50,000 in Immuno-enhancer	
	Goat anti mouse IgG-HRP (sc-2005)	Santa Cruz Biotechnology	1/50,000 in 10% Block Ace - TBST	
	Goat anti rabbit IgG-TR (sc-2780)	Santa Cruz Biotechnology	1/1000 in 10% Block Ace - PBS(-)	
	Goat anti mouse IgM-PE (sc-3768)	Santa Cruz Biotechnology	1/1000 in 10% Block Ace - PBS(-)	
